# Algae: Study of Edible and Biologically Active Fractions, Their Properties and Applications

**DOI:** 10.3390/plants11060780

**Published:** 2022-03-15

**Authors:** Olga Babich, Stanislav Sukhikh, Viktoria Larina, Olga Kalashnikova, Egor Kashirskikh, Alexander Prosekov, Svetlana Noskova, Svetlana Ivanova, Imen Fendri, Slim Smaoui, Slim Abdelkafi, Philippe Michaud, Vyacheslav Dolganyuk

**Affiliations:** 1 Institute of Living Systems, Immanuel Kant Baltic Federal University, A. Nevskogo Street 14, 236016 Kaliningrad, Russia; olich.43@mail.ru (O.B.); stas-asp@mail.ru (S.S.); surinac@mail.ru (V.L.); kalashnikova_14@bk.ru (O.K.); egorkah@mail.ru (E.K.); svykrum@mail.ru (S.N.); dolganuk_vf@mail.ru (V.D.); 2 Laboratory of Biocatalysis, Kemerovo State University, Krasnaya Street 6, 650043 Kemerovo, Russia; a.prosekov@inbox.ru; 3 Natural Nutraceutical Biotesting Laboratory, Kemerovo State University, Krasnaya Street 6, 650043 Kemerovo, Russia; 4 Department of General Mathematics and Informatics, Kemerovo State University, Krasnaya Street 6, 650043 Kemerovo, Russia; 5 Laboratoire de Biotechnologie Végétale Appliquée à l’Amélioration des Cultures, Faculté des Sciences de Sfax, Université de Sfax, Sfax 3038, Tunisia; imen.fendri@fss.usf.tn; 6 Laboratoire de Microorganismes et de Biomolécules, Centre de Biotechnologie de Sfax, Route Sidi Mansour Km 6 B.P. 117, Sfax 3018, Tunisia; slim.smaoui@yahoo.fr; 7 Laboratoire de Génie Enzymatique et Microbiologie, Equipe de Biotechnologie des Algues, Ecole Nationale d’Ingénieurs de Sfax, Université de Sfax, Sfax 3038, Tunisia; slim.abdelkafi@enis.tn; 8 Institut Pascal, Université Clermont Auvergne, CNRS, Clermont Auvergne INP, 63000 Clermont-Ferrand, France; 9 Department of Bionanotechnology, Kemerovo State University, Krasnaya Street 6, 650043 Kemerovo, Russia

**Keywords:** macroalgae, microalgae, biological activity, antioxidant, functional nutrition

## Abstract

The beneficial properties of algae make them perfect functional ingredients for food products. Algae have a high energy value and are a source of biologically active substances, proteins, fats, carbohydrates, vitamins, and macro- and microelements. They are also rich in polyunsaturated fatty acids, proteins, mycosporine-like amino acids, polysaccharides, polyphenols, carotenoids, sterols, steroids, lectins, halogenated compounds, polyketides, alkaloids, and carrageenans. Different extraction parameters are used depending on the purpose and the substances to be isolated. In this study, the following parameters were used: hydromodule 1:10 and an extraction duration of 1–2 h at the extraction temperature of 25–40 °C. A 30–50% solution of ethanol in water was used as an extractant. Algae extracts can be considered as potential natural sources of biologically active compounds with antimicrobial activity and antiviral properties. The content of crude protein, crude fat, and carbohydrates in *U. Prolifera*, *C. racemosa* var. *peltata* (*Chlorophyta*), *S. oligocystum* and *S. fusiforme* (SF-1) was studied. It was found that *C. muelleri* (*Bacillariophyta*), *I. galbana* (*Haptophyta*), and *T. weissflogii* (*Bacillariophyta*) contain about 1.9 times more omega-3 than omega-6 fatty acids. *N. gaditana* (*Ochrophyta*), *D. salina* (*Chlorophyta*), *P. tricornutum* (*Bacillaryophyta*) and *I. galbana* (*Haptophyta*) extracts showed inhibitory activity of varying intensities against *E. coli* or *P. aeruginosa*. In addition, algae and algae-derived compounds have been proposed to offer attractive possibilities in the food industry, especially in the meat sector, to evolve functional foods with myriad functionalities. Algae can increase the biological activity of food products, while the further study of the structure of compounds found in algae can broaden their future application possibilities.

## 1. Introduction—Characteristics of Algae

Algae are prokaryotic (cyanobacteria) or eukaryotic photoautotrophic organisms that can convert nitrogen and phosphorus from the environment into biomass using light, CO_2_, and water. The resulting biomass can subsequently be fractionated into various bioproducts using a suitable process [1]. Algae can use sunlight for photosynthesis or can exist as mixotrophs or facultative heterotrophs [2]. Some of the latter lost the ability to photosynthesize and turned into obligate heterotrophic parasites such as *Plasmodium* and *Toxoplasma* (*P. malariae*, *P. knowlesi*, *P. falciparum*, *P. vivax*, and *P. ovale*) [3].

Algae have several reproductive strategies and can be unicellular organisms or have complex multicellularity [3]. Algae can be divided into micro- and macroalgae. Microalgae (microphytes) are represented by green (*Chlorophyta*), blue-green (*Cyanobacteria*), yellow-green (*Ochrophyta* и *Xanthophyta*), and golden (*Ochrophyta* и *Chrysophyta*) algae, and diatoms (*Bacillaryophyta*). Macroalgae or simple algae include red (*Rhodophyta*), green (*Chlorophyta*), and brown algae (*Ochrophyta*) [4].

Macroalgae are mainly found in the marine environment [5]. They are available naturally or can be cultivated in large areas of the seaside. Algae use natural nutrients available in the sea for their growth [6].

The main primary metabolites of algae are lipids, proteins, carbohydrates, and water (Table 1) [7,8]. Chlorophylls, cytochromes, nucleotides, and compounds that are intermediates in various metabolic reactions are also primary metabolites [8]. The biochemical composition of microalgae makes them suitable for producing various compounds. The ratio of primary metabolites depends on the type of algae and the conditions of their cultivation [6]. For example, *S. maxima* (*Cyanobacteria*) is an excellent protein source (60–71% by weight) [9], *P. cruentum* (*Cyanobacteria*) is a rich source of carbohydrates (40–60%) [9], and *S. dimorphus* (*Chlorophyta*) contains 40% lipids [10].

In addition to primary metabolites, macroalgae contain secondary metabolites, which are substances that are not involved in the main metabolism and may be specific to one or more algae species. Agar, alginate, fucoidan, ulvan, laminarin, starch, cellulose (1,4-β-D-glucan), pectin substances β-D-mannuronic and α-L-guluronic, and carrageenan are examples of secondary metabolites of algae [7]. Algae produce a large number of secondary metabolites during their life cycle, making them an important natural source of these bioactive compounds [15].

Algae are rich sources of biologically active compounds with antiviral, antitumor, and anti-inflammatory properties, and they are also sources of plant growth stimulators or antioxidant agents [16]. Components of microalgae help maintain the health of the cardiovascular system and exhibit anti-inflammatory, anticoagulant, antiviral, antibacterial, antifungal, and other properties. Components of microalgae are used to strengthen the immune system, lower blood cholesterol levels, and are effective against hypercholesterolemia. The active components of algae can remove harmful elements from the human body and also have ulcer- and wound-healing properties. Microalgae extracts can increase hemoglobin concentration, reduce the level of sugar in the blood, and exhibit analgesic, bronchodilatory, and hypotensive activities [17]. As a result, more and more attention is being paid to applying algae in the pharmaceutical, cosmetic, and food industries [16]. The main biochemical compounds of algae include carbohydrates, proteins, lipids, and minerals (micro- and macroelements) [7,18]. The carbohydrates present in algae are polysaccharides, which can be divided into matricial (agar, alginate, fucoidan, and ulvan), storage (laminarin and starch), and fibrous (cellulose) polysaccharides. Phycobiliproteins are pigment proteins that can be used in a variety of applications. Minerals such as micro- and macroelements are present in large quantities in algae [7].

The possible applications of various algae are quite extensive: energy production [19,20], the bioremediation of industrial and domestic wastewaters [21,22,23,24,25], the removal of carbon dioxide from industrial flue emissions via algae biofixation [26,27], the production of ethanol or methane [28], feed for livestock, raw material for pyrolysis [4], organic fertilizer [26], or biostimulants in agriculture [29]. In addition, algae are used as raw materials to produce third-generation biofuels [30,31,32,33,34,35,36,37]. *C. reinhardtii*, *C. vulgaris*, *D. salina*, and diatoms are the most studied microalgae species for biofuel production [38].

Gelling, thickening, and stabilizing substances such as agar, alginate, and carrageenan are obtained from algae [39]. Algae have undeniable advantages in various fields of application [40].

The interest in foods from whole algal biomass is attributed to reports of high protein content and health benefits [41,42]. Algae can be used as functional ingredients to enhance the nutritional value of foods [43]. However, most algae have thick cell walls, which makes it difficult to extract nutrients and biologically active components. In this regard, algae processing requires a suitable technology for destroying cells without the denaturation of active components [42].

In the life process, algae produce many secondary metabolites, which makes them an important natural source of these bioactive compounds [15]. The addition of preparations obtained from algae allows additional beneficial properties to be imparted to food products in addition to the traditional nutritional value [8]. Thus, functional nutrition with algae ingredients has a beneficial effect on human health, improving well-being and quality of life, as well as reducing the risk of diseases depending on the use of appropriate technologies to lyse cells and extract active products in mild conditions.

The growing interest in algae application in food has led to a lot of research and the accumulation of data on food from algae. This field requires the systematization and generalization of the available results. This review discusses the nutritional and bioactive components of algae, their chemical characteristics and biological properties, as well as methods of their extraction. The use of algae as bioactive ingredients for functional nutrition (organization of the daily diet and food intake to ensure that the human body receives the required amount of minerals, vitamins, amino acids, carbohydrates, and proteins), as well as the problems arising in the industrial production of algae components, are also considered.

This study aimed to investigate the edible and biologically active fractions of algae, their composition and properties, as well as the use of algae in the food, pharmaceutical, chemical, and agricultural industries, as well as for environmental cleaning. Thus, the study aimed to describe the biologically active fractions of algae, their properties, and the applications. The need for such research stems from the discovered information gaps, inconsistencies in research information (differing research results), the presence of differing scientific opinions, and the identification of various leading trends in research.

## 2. Microalgae

### 2.1. Microalgae Nutritional Composition

Microalgae have long been used as food in Asian countries such as China, Japan, and Korea, as they have many beneficial properties [44]. Algae are an excellent source of proteins, fats, and carbohydrates and contain many vitamins and macro- and microelements [45] and have a high energy value [46].

The microalgae composition varies depending on the growing conditions. For example, the dynamics of the total content of proteins, carbohydrates, and lipids in green microalgae (*S. obliquus*, *D. armatus*, *D. subspicatus*, *S. obtusus*, *M. contortum*, and *S. gracile*) in response to the effect of short-term temperature fluctuations (±10 °C) were investigated [47]. Sharp temperature drops led to a reversible redistribution of the content of biochemical components in the cells of green microalgae. This phenomenon is species-specific and depends on the age of the culture. A decrease in temperature led to an increase in carbohydrate content, and the inverse led to a decrease in lipid content.

Filamentous cyanobacterium *A. platensis* (also known as *S. platensis*) and various commercial species of the unicellular green alga *C. vulgaris* contain up to 70% dry protein. These microalgae and notably *C. muelleri* also have an amino acid profile comparable to that of eggs [48,49]. All essential amino acids were found in the consortium of *C. vulgaris* and *S. obliquus* microalgae [48,49].

Microalgae are rich in lipids mainly composed of esterified saturated and unsaturated fatty acids [50,51]. A study [52] demonstrated high lipid content in *C. muelleri* (*Bacillariophyta*) and *I. galbana* (*Haptophyta*). *T. weissflogii* (*Bacillariophyta*) also contained lipids in its composition but in smaller quantities. The microalgae consortium consisting of *C. vulgaris* and *S. obliquus* contained saturated fatty acids with chain lengths from 14 to 18, mono-, di-, and polyunsaturated fatty acids. Omega-3 content was about 1.9 times higher than the amount of omega-6 fatty acids [50]. Fatty acids such as palmitoleic, oleic, palmitic, cis-5,8,11,14,17-eicosapentaenoic, arachidonic, and g-linolenic acids were extracted from samples of the microalgae *N. salina* (*Ocrophyta*) [53].

Microalgae are rich in exopolysaccharides [54,55,56]. Cyanobacterial exopolysaccharides have some structural features compared to polymers produced by other microorganisms, such as the presence of one or two uronic acids, the presence of sulfate groups—a feature unique to bacteria, but distinguishing exopolysaccharides produced by archaea and eukaryotes [54]. A capsular, water-insoluble polysaccharide with a complex structure was isolated from the blue-green algae *M. laminosus* [57]. It contained a repeating polyglycan unit. *P. marinum*, *P. purpureum*, and *R. violacea* are a source of sulfated exopolysaccharides [55,56,58].

In view of the abovementioned research, microalgae are an excellent source of nutrients.

### 2.2. The Potential Health Benefits of Microalgae

#### 2.2.1. Biologically Active Components of Microalgae

A wide variety of compounds synthesized by different pathways of algae metabolism provide promising sources of polyunsaturated fatty acids, polyphenols, sterols and steroids, carotenoids, polysaccharides, lectins, mycosporine-like amino acids, proteins, halogenated compounds, polyketides, alkaloids, alginic acid, and carrageenans [59,60,61].

Preparations based on microalgae have therapeutic properties such as antitumor, anti-inflammatory, anticoagulant, antiviral, antibacterial, antifungal, wound healing, and others [62,63,64,65,66,67]. Microalgae components are used to strengthen the immune system and lower blood cholesterol levels, are beneficial for the health of the cardiovascular system, effective against hypercholesterolemia, and can remove harmful elements from the human body. Microalgae extracts increase hemoglobin concentration and lower blood sugar.

**Carotenoids and phenolic compounds.** Carotenoids, terpenoid pigments derived from tetraterpenes (C40), are essential pigments in algae [68]. For example, fucoxanthin exhibits biological activity in various model systems, providing antioxidant, anticancer, antidiabetic, anti-obesity, anti-inflammatory, hepatoprotective, antiangiogenic, antimalarial effects. Fucoxanthin is safe for human consumption and, therefore, can be used as a bioactive molecule for the prevention and treatment of diseases in humans [69].

Astaxanthin is another important pigment in microalgae, which is of particular interest because of its potent antioxidant activity [70]. The extraction of this compound from the *C. zofingiensis* (*Chlorophyta*) biomass was successfully explored [71]. Astaxanthin ester dominated free astaxanthin regardless of culture conditions and time points. However, a higher proportion of ester was observed under stress conditions. Another study addressed the production of astaxanthin as a by-product in biodiesel production from microalgae [72].

Fucoxanthin, astaxanthin, lutein, and zeaxanthin contained two alcohol OH groups at different ends of the molecule, but the molecules themselves had different configurations, whereas canthaxanthin and β-carotene were characterized by the absence of OH groups.

Microalgae *H. pluvialis*, *C. zofingiensis*, and *D. disociatus* (*Chlorophyta*) are potential sources of canthaxanthin, a pigment with antioxidant and immunomodulatory properties [73,74]. An extract of *D. subspicatus* contained lutein [75]. It has antioxidant potential and plays a significant role in age-related infant brain development, age-related macular degeneration, and cancer [76]. Some microalgae species (*C. fusca*, *C. citroforme*, *T. intermedium*, *S. almeriensis*, *D. protuberans*, and *A. protothecoides*) are potential lutein sources, as they produce about 5 g∙kg^−1^ biomass mainly in the form of free lutein [76].

Microalgae can also be rich in phenolic compounds. Significant phenolic acid (mainly gallic acid) content with antioxidant activity was identified in *I. galbana* (*Haptophyta*) extracts [77].

**Minerals and vitamins.** It was noted that among microalgae, *C. vulgaris*, *H. pluvialis*, *D. salina* (*Chlorophyta*), and *S. maxima* (*Cyanobacteria*) were rich in iodine, potassium, iron, magnesium, and calcium [78]. The human body absorbs algal iron more easily than that from higher terrestrial plants due to the pigment phycocyanin, which forms soluble complexes with iron and other minerals during digestion.

Microalgae contain vitamins [79] such as B_12_ [80] or K [81]. *C. vulgaris*, *H. pluvialis*, *D. salina*, and *S. maxima* are rich in vitamins A, C, B_1_, B_2_, B_3_, and B_6_ [78].

Some studies have suggested that vitamin B_12_ from algae is not bioavailable to humans [82]. However, other studies have found that *C. vulgaris* contains the active form of vitamin B_12_. Studies in rats with B_12_ anemia and a nutritional analysis of vegan children have shown that consuming *C. vulgaris* helps prevent B_12_ deficiency [83,84]. Thus, algae products are one vegetarian alternative to the cobalamin source in one’s diet [85].

#### 2.2.2. Antimicrobial and Antiviral Activity of Microalgae

The literature points to the antimicrobial activity of microalgae extracts. The problem of maximizing the extraction of biologically active substances while preserving their properties was solved while developing a technology for producing extracts from microalgae. When selecting rational extraction conditions, the following parameters were studied: hydromodule (ratio of raw materials: extractant), duration and temperature of extraction, and type of extractant. The following parameters were used: hydromodule 1:10 and an extraction duration of 1–2 h at the extraction temperature of 25–40 °C. A 30–50% solution of ethanol in water was used as an extractant [86]. Extracts of the blue-green algae *A. oryzae*, *O. limosa*, and *S. ocellatum* were tested for their antimicrobial activity against pathogenic human fungal and bacterial strains (*B. subtilis*, *M. luteus*, *S. aureus*, *E. coli*, *K. pneumoniae*, *P. aeruginosa*, and *S. marcescens*) using the disc diffusion method. The acetone extract of *A. oryzae* was the most active against the tested fungal and bacterial strains, while it showed the maximum antimicrobial activity against *S. marcescens* and *C. albicans* [86].

A review [86] studied the antimicrobial activity of ethanol extracts of several microalgae species against pathogenic and opportunistic microorganisms. *T. suecica* extract showed an inhibitory effect against three studied bacteria [87].

It was found that *N. gaditana* (*Ochrophyta*), *D. salina* (*Chlorophyta*), *P. tricornutum* (*Bacillaryophyta*), and *I. galbana* (*Haptophyta*) extracts had inhibitory activity of varying intensities against *E. coli* or *P. aeruginosa. N. gaditana* extract also inhibited *S. aureus. C. muelleri* (*Bacillariophyta*) and *C. vulgaris* (*Chlorophyta*) extracts showed no antimicrobial activity. The extracts of all studied algae suppressed *C. albicans*. However, *M. gaditana* was most active. *A. niger* was found to be resistant to the action of all extracts. The observed antimicrobial activity was associated with the content of fatty acids, carotenoids, and phenolic compounds in the extracts.

The antiviral activity of microalgae is also known, due to the presence of sulfated polysaccharides in their composition [88,89].

Thus, microalgae extracts can be considered a potential natural source of biologically active compounds with antimicrobial and antiviral activities. However, more in vivo studies are needed before algae-based antimicrobial agents can be used in humans.

It was found that macroalgae also have antimicrobial and antiviral activities [90,91].

## 3. Macroalgae

### 3.1. Nutritional Value of Macroalgae

As a food product, microalgae provide high nutritional value but with a reduced calorie content [44,92]. In addition to microalgae, macroalgae are an excellent source of proteins, fats, and carbohydrates (Table 1) and contain many vitamins and macro- and microelements [11,93,94,95]. At the same time, the algae composition can vary depending on the time and place of collections [11,13,14,96].

The protein content in different algae groups varies greatly. Among marine macroalgae, red and green algae (e.g., *P. vulgaris* (*Laverbread*), *P. tenera* (*Nori*), *P. palmata* (*Dulse*), and *U. lactuca* (*Sea lettuce*)) often contain high levels of protein (as a percentage of dry weight) in contrast to lower levels in most brown algae [7]. High contents of protein and amino acids were found in macroalgae *U. pinnatifida* (*Ochrophyta*) composition [46]. *U. prolifera* (*Chlorophyta*) has been studied as a food source with low fat and high protein contents [11].

The edible green alga *C. racemosa* var. *peltata* contained 40.07% essential amino acids [97]. Eighteen amino acids were found in *S. oligocystum* (aspartic acid, glutamic acid, serine, glycine, arginine, alanine, tyrosine, cysteine, proline, histidine, threonine, valine, methionine, isoleucine, phenylalanine, tryptophan, leucine, and lysine). Their amounts varied depending on the season [13], reaching the highest values in May.

Algae are rich in fatty acids. Among macroalgae, *S. fusiforme* has a high content of polyunsaturated fatty acids including eicosapentaenoic, α-linolenic, and arachidonic acids [14]. The main fatty acids found in *S. oligocystum* were palmitic, oleic, and arachidonic acids. These algae had a surprisingly high saturated fatty acid composition. For example, palmitic acid accounted for 37–45% of all fatty acids [13].

Carbohydrates are a common algae component [97]. Algae polysaccharides can selectively increase the activity of certain populations of beneficial bacteria and stimulate the production of functional metabolites by the gut microbiota. In addition, they can stimulate a number of biological activities such as anticancer, antioxidant, immunomodulatory, and antidiabetic ones. Algae polysaccharides are not absorbed by human digestive enzymes. They are resistant to digestion in the upper intestinal tract and are subsequently fermented in the colon [98]. High carbohydrate contents were measured in the red algae *C. crispus*, *M. stellatus*, and *G. pistillata* [99]. It was established, for example, that the brown algae *H. elongata* and *L. ochroleuca* contain high contents of a sulfated polysaccharide, fucoidan [46]. Fucoidans extracted from algae have a complex structure, which depends on the type of algae, the part of the plant, and the extraction method used [100]. Depending on their structures, they can have a number of health benefits, such as anticancer, antioxidant, and antiviral activities [101]. In addition to fucoidan, other polysaccharides, such as alginates, were also found in *S. fusiforme* and other brown algae [50].

It was shown that the red algae *C. crispus*, *M. stellatus*, and *G. pistillata* have high energy values for living organisms [102].

### 3.2. Some Bioactive Components of Algae

Many bioactive products derived from algae are used in the pharmaceutical and food industries. The biologically active substances of algae are mainly determined using gel permeation chromatography (GPC). GPC is a liquid chromatography technique in which a polymer in solution is separated into separate chains depending on their size (and not on chemical properties) and by the presence of functional groups (using affinity chromatography, a type of ligand chromatography). The latter was based on the reaction of the interaction of separated impurities with a ligand bound to an inert carrier, seharose. In the case of affinity chromatography, biologically active substances (proteins and enzymes) that enter into a specific biochemical interaction with a ligand (also, as a rule, organic) were used as impurities. Gas–liquid chromatography, IR, proton nuclear magnetic resonance, and 13C NMR spectroscopy, high-performance size exclusion chromatography, and the C-PC method (analysis method with the isolation of a pigment–protein complex from the family of light-harvesting phycobiliproteins—C-phycocyanin) are also used to determine the chemical composition of algae [103,104].

**Carotenoids.** Like microalgae, macroalgae are rich in the carotenoid fucoxanthin, which can have antioxidant, antitumor, antidiabetic, anti-inflammatory, hepatoprotective, antiangiogenic, and antimalarial effects [69].

Pigments β-carotene and zeaxanthin, which macroalgae are rich in, also exhibit antioxidant properties. The dependence of the β-carotene and zeaxanthin concentrations in red algae *P. yezoensis* on environmental conditions was demonstrated [102]. When extracting carotenoids, it is necessary to select suitable methods of purification from interfering components, which will not change the structure of the active component. One of these methods is the purification of carotenoids from chlorophylls using activated carbon [105].

**Phenolic compounds.** Phenolic compounds of algae also have biological activity, such as antioxidant, antiproliferative, antiobesity, and antidiabetic activities [106]. For example, the antioxidant and antidiabetic effects of phlorotannins isolated from *C. compressa* were detected and quantified [107]. Flavonoids extracted from the Libyan brown algae *C. compressa* and *P. pavonica* exhibited bactericidal activity against pathogenic bacteria isolated from meat, meat products, milk, and dairy products. The best spectrum of bactericidal action was shown by flavonoids extracted from *C. compressa* [95].

Derivatives of phenolic acid, phlorotannin, catechin, hydroxybenzoic acid, coumaric acid, and gallocatechin, were identified in the extracts from brown algae *A. nodosum*, *L. japonica*, *L. trabeculata*, and *L. nigrescens* [108].

One study [109] identified various phlorotannins, phenolic acids, and flavonoids (mainly acacetin derivatives) in *A. nodosum*, *B. bifurcata*, and *F. vesiculosus* extracts. The content of phenolic compounds in algae changes under various abiotic and biotic factors [110]. Phenolic acids can be found in free form in algae extracts, but they are more commonly found in the form of conjugates. For example, in the form of esters. Gallic and ellagic acids can be esterified with glucose or another monosaccharide [111]. Preparative chromatography methods such as high-performance liquid chromatography (HPLC) or thin-layer chromatography (TLC) allow for the isolation and purification of individual phenolic compounds in pure forms [111] without changing their structure.

The chemical diversity of *U. reticulata* (*Chlorophyta*), *S. wightii*, and *G. verrucosa* (*Rhodophyta*) was demonstrated [112]. Over 30 metabolites were found, and steroids and fatty acids were dominant.

**Minerals and vitamins.** Algae are high in minerals. Sea algae are rich in iodine, so they can be potential candidates for the production of drugs for the prevention of many diseases caused by iodine deficiency (for example, endemic goiter, Grave’s disease, and hyperthyroidism) [113]. Significant Ca, Mg, K, Cu, Fe, and Se contents were found in *S. fusiforme* and *S. oligocystum*, collected from May to August [13,14]. *S. oligocystum*, collected in February, had higher Na, I, and Zn contents [13]. *L. japonica* contain 13 times more calcium than milk [114].

Macroalgae produce or store a wide variety of vitamins [115]. Algae are valuable candidates for preventing diseases associated with iron and vitamin B_12_ deficiency (alimentary anemia) and vitamin A (xerophthalmia). Algae are rich in antioxidant vitamins C and E. Vitamin C prevents scurvy, and vitamin E helps to manage neurological problems caused by poor nerve conduction and anemia caused by oxidative damage to red blood cells [114,115]. Macroalgae *P. vulgaris* and *P. palmata* demonstrated high vitamin B_12_ content [46]. Retinol, α-tocopherol, and ergocalciferol were identified in *C. barbata* [116].

### 3.3. Antimicrobial and Antiviral Activities

Due to the significant content of various biologically active components, algae can exhibit antimicrobial, antimycotic, and antiviral activities.

**Antimicrobial and antimycotic activity.** There is a significant amount of research on the antimicrobial and antimycotic activity of macroalgae extracts. One study [116] demonstrated the high antimicrobial activity of red alga *G. doryphora* extracts (in methanol, ethanol, and ethyl acetate) against *B. subtilis*, *E. faecalis*, *S. aureus*, *E. coli*, *P. aeruginosa*, and *C. albicans* (yeast strain). The antimicrobial activity was evaluated in vitro using the well-cut diffusion technique. The fresh extract was found to be more effective against all tested organisms than the dried material, except for the ethyl acetate and ethanol extracts against *B. subtilis*.

Sirbu et al. [117] studied the antimicrobial activity of the green algae *U. lactuca*, *E. intestinales*, and *C. vagabund* against *E. coli* and *S. aureus*. The analysis method used was the nutrient agar well diffusion method. It was shown that the sites of inhibition in *E. coli* are higher than in *S. aureus*. The algae *U. lactuca* (*Chlorophyta*), *D. spiralis*, and *J. rubens* (*Rhodophyta*) were tested for activity against Gram-positive (*S. pyogenes* and *M. luteus*), Gram-negative (*S. flexneri* and *V. cholerae*) bacteria and fungi (*C. albicans* and *A. niger*). *M. luteus* was the most sensitive pathogen. Among all the studied extracts (water, methanol, ethanol, chloroform, acetone, ethyl acetate, and hexane), the chloroform extract of *D. spiralis* was the most active against the studied pathogens. Water extracts were not active against all selected pathogens [118].

The data from the studies mentioned above are summarized in Table 2. It indicates the data on the antimicrobial and antifungal activity of microalgae for comparison. The table shows that many algae are active against *C. albicans*. Compared to microalgae, all of the mentioned macroalgae showed activity against *A. niger*, while none of the indicated microalgae showed activity against this type of microorganism. All of the mentioned macroalgae extracts demonstrated activity against *E. coli*, and the majority of microalgae extracts also showed activity against *E. coli*, except for *O. limosa*, *S. ocellatum* (*Cyanobacteria*), *C. muelleri* (*Bacillariophyta*), and *C. vulgaris* (*Chlorophyta*). All the listed macroalgae extracts were active against *S. aureus*. However, among microalgae, only *A. oryzae*, *T. suecica* (*Chlorophyta*), and *N. gaditana* were active. The different ratios of total lipids and polyunsaturated fatty acids in macro- and microalgae can explain differences in their antimicrobial activity. The higher this ratio, the more active the macro- or microalgae extract is. Macroalgae have the most fungicidal activity, and they also contain a lot of triacylglycerols, glyceroglycolipids, phospholipids, fucoxanthin, and other biologically active substances that adversely affect the higher mold fungi *A. niger* [119,120].

**Antimicrobial activity of endophytic fungi isolated from algae.** The antimicrobial effect of algae may be due to their active substance contents but also to secondary metabolites formed by endophytic fungi present in these plants. For example, the antimicrobial properties of fungi extracts isolated from the brown algae *P. pavonica* against *S. aureus*, *E. coli*, and *C. albicans* were demonstrated [122]. Agar diffusion was used as an analysis method. One of the fungal isolates, identified as *T. harzianum*, showed the greatest activity against the above microorganisms. Twenty-seven endophytic fungi were isolated from seven macroalgae (*D. divaricata*, *G. lauris*, *G. salicornia* (*Rhodophyta*), *P. minor*, *U. lactuca*, *S. oligocystum*, and *S. polycystum*). The cross-band method was performed to screen for antibacterial agents. Thirteen of these fungi had positive antibacterial activity against six pathogenic human bacteria, such as *S. aureus*, *S. marcescens*, *S. typhi*, *S. dysenteriae*, *E. coli*, and *K. pneumoniae* [123]. The fungus *T. viride* demonstrated strong broad-spectrum antibacterial activity. Thus, endophytic fungi isolated from macroalgae can be a source of antimicrobial compounds.

**Antiviral activity.** The antiviral activity of macroalgae is often determined by the content of polysaccharides in their composition. Carrageenans, ulvans, fucoidans, agars, and alginates have strong antiviral properties [124,125]. It was reported that some of the sulfated polysaccharides could be used to prevent and treat COVID-19 [126,127]. In vitro studies demonstrated that iota-carrageenan can inhibit SARS-CoV-2 [128,129]. Fucoidan prevents SARS-CoV-2 from entering the cell by binding to S-glycoprotein [129,130]. Fucoidan and other highly sulfated polysaccharides were tested in vitro using surface plasmon resonance (SPR) to measure the binding affinity for the SARS-CoV-2 S protein [130].

There are currently no algae-based antimicrobial, antifungal, or antiviral drugs registered. In vivo studies are required to extrapolate use to humans. Nonetheless, macroalgae are promising candidates for further research aimed at their future use in the pharmaceutical industry.

### 3.4. Macroalgae as Bioactive Ingredients for Functional Food

According to the National Academy of Sciences’ Institute of Medicine, “functional foods are foods that encompass potentially healthful products, including any modified food or food ingredient that may provide a health benefit beyond the traditional nutrients it contains” [131].

#### 3.4.1. Algae in the Food Industry

While increasing the worldwide market of functional foods, in parallel, there is growing interest in the discovery of new functional food ingredients from different natural sources [132,133,134,135]. Thus, in recent years, the opportunity of using algae-derived compounds for innovative functional food products has become of great interest. In the food industry segment, the major and most commonly used hydrocolloids from marine algae are: agars, alginates, and carrageenans.

##### Agar

Agar is principally found in the matrix cell of seaweeds of the *Gelidiales* (*Gelidium* and *Pterocladia*) and *Gracilariales* (*Gracilaria* and *Hydropuntia*) orders. Its abundance and its easy exploitation allow agar production from *G. tenuistipitata*, which is economically important [136].

Agar is a phycocolloid composed of agarose (a linear polysaccharide) and a heterogeneous mixture of smaller molecules (agaropectin). A generally recognized as safe (GRAS) food additive in USA and a food additive authorized in Europe (E406), agar cannot be digested in the gastrointestinal tract because humans lack α/β-agarases. Nonetheless, it can be metabolized by intestinal bacteria to d-galactose [137]. Agar is an effective gelling agent, able to form a brittle, firm, and thermally reversible gel at low concentrations [138]. Remarkably, agarose is the main gelling agent in agar. In this regard, along its linear chains of agarose with repeating units, agar gel is formed by hydrogen bonds between the adjacent d-galactose and 3,6-anhydro-l-galactose. Ninety percent of produced agar is used in the food industry for its gellifying properties. It is used in in culinary, food, and confectionery industries as the gelling agent for producing Asian traditional dishes, canned meats, confectionery jellies, and aerated products such as marshmallows, nougat, and toffees [138,139]. Served as a food additive, agar is routinely used to produce foods that need heating before consumption, such as cake, sausage, roast pork, and bacon [140]. In order to substitute fat in whipped products, agar fluid gels can be used for making foams at high stability [141]. On the other hand, the remaining 10% is employed as a thickening component of media for the culture of bacteria tissue, cells, filamentous fungi, and yeast [142].

Agar can be defined as a hydrophilic colloid extracted from some algae of the *Rhodophyceae* class. It does not dissolve in cold water, but it does dissolve in boiling water. The 1.5% solution is clear, and when it cools down to 34–43 °C, it forms a solid gel that does not dissolve again at a temperature below 85 °C. It is a mixture of polysaccharides, the main monomer of which is galactose. These polysaccharides can be sulfated to very different degrees, but to a lesser extent than in carrageenan. For this reason, the ash content of agar is lower than that of carrageenan, furcelleran, and other polysaccharides. Agar is characterized by a maximum ash content of 5.0%, although it is usually maintained within the range of 2.5–4.0% [143].

##### Alginates

Present as a blended salt of Na and/or Na, Ca, and Mg in brown macroalgae, alginates are polymers of consecutive mannuronate and guluronate (1,4) covalently connected together in various blocks [144]. The principal marketable sources of alginate are marine brown algae, and notably, those belonging to *L. japonica*, *A. nodosum*, and *L. trabeculata* genus [144]. Similar to agar, in the food industry, alginates are usually employed for gelling, thickening, stabilizing, and film-forming applications. Interestingly, contrary to other hydrocolloids, alginates are special in their cold solubility, which permits the manufacture of heat/temperature-independent non-melting gels, cold-setting gels, and freeze–thaw-stable gels [38,145]. Technically, for alginate gel formation, the addition of cations such as Ca^2+^ is desired. In this vein, only guluronate blocks and occasionally mannuronate/guluronate blocks can react with Ca^2+^ to form alginate gels [146]. It should be noted that commanding alginates–Ca interaction would confer shear-irreversible and heat-stable features on cold-setting gels [38,145]. In the food industry, solely Na alginate is predominantly used in foods as gel or as a viscosity regulator [38,145]. For the majority of Na alginate applications (e.g., custards, bakery fillings, structured fruits, structured vegetables, aerated confectioneries, and structured meat products), internal setting under a governed status is crucial [144]. Moreover, Na alginate can be used as a thickening and structuring agent in low-fat margarine and spread products and can also be used to control the melting behavior of ice cream and reformed foods such as onion rings and olive fillings [144,147]. Likewise, alginate is exploited as a stabilizer of beer foam [143].

Alginate is a linear polysaccharide in which β-D-mannuronate (M) and α-L-guluronate (G) are covalently (1–4) linked in different sequences. α-L-Guluronate is the C5 epimer of β-D-mannuronate. Monomers of uronic acid are linked into a polymannuronate block (polyM block), a polyguluronate block (polyG block), and a random copolymer (polyMG block) [148].

As a hydrocolloid cryoprotectant, alginates impart different cryoprotective effects to food products depending upon their solubility, water-holding capacity, rheological properties, and synergistic effect with other ingredients during freezing and frozen storage. For instance, [149] reported the usefulness of alginate in retaining the texture and sensory acceptability of pre-cut carrots during frozen storage. Likewise, the addition of sodium alginate to corn starch, sucrose, or water mixtures minimized the structural damage to the gel/paste after slow freezing and during frozen storage.

Lee et al. [150] concluded that sodium alginate was effective in syneresis reduction in sweet potato starch gel after five freeze–thaw cycles and storage at −18 °C for 20 h followed by 25 °C for 4 h.

##### Carrageenan

The initial raw material for the production of carrageenan, an ionic polysaccharide, is *C. crispus*, commonly known as “Irish moss” [151]. *K. alvarezii* and *E. denticulatum* are also used [152]. With diverse structural specificities, carrageenans are sulfated polysaccharides. There are three main commercial kinds of carrageenan: kappa (which has one sulfate per disaccharide), iota, and lambda (which comprise two and three sulfates per disaccharide, respectively) [153]. These carrageenans differ in the degree of sulfation and consequently have diverse gel strengths, textures, solubilities, melting and setting temperatures, syneresis, and synergy properties [145,153,154]. In the presence of potassium ions (K^+^), kappa-carrageenan forms a strong and rigid gel and can react with dairy proteins. In contrast, iota-carrageenan forms a soft and elastic gel in the presence of Ca^2+^ ions [49,51]. However, lambda-carrageenan is a pure thickener and does not gel [155].

Employed for decades in the food industry, carrageenan is labeled as a GRAS by the Food and Drug Administration (FDA); additionally, carrageenan and semi-refined carrageenan are food additives (E-407 and E407a, respectively) permitted by the European Food Safety Authority (EFSA). Carrageenan is widely used in dairy products, such as cheese and chocolate milk products, to confer thickening, gelling, stabilizing, and strong protein-binding properties [152]. Due to its extraordinary faculty to join milk proteins, carrageenan, at low levels, was utilized in dairy products. This hydrocolloid was able to keep milk solids in suspension, therefore stabilizing them. Another domain in the application of carrageenan (mainly produced by *Eucheuma*) is the meat industry. Due to its water retention characteristics, it is used in the production of hamburgers, ham, seafood, and poultry products. Carrageenan is also used in aqueous gels, such as jelly-candies, fruit gels, juices, and marmalade [140,143]. For instance, Atashkar et al. [155] assessed the impact of the addition of κ-carrageenan at 0.5%, 1.0%, and 1.5% on texture characteristics of sausages formulated with 70% fat reduction and stored at 4 °C over 30 days. The results displayed that the partial fat substitution with κ-carrageenan lead in a decrease in hardness and chewiness and a partial increase in gumminess and springiness.

Carrageenans are high-molecular-weight, sulfated D-galactans consisting of repeating disaccharide units with alternating 3-linked β-D-galactopyranose (G-units) and 4-linked α-galactopyranoses (D-units) or 3,6-anhydro-α-galactopyranose (AnGal units) [156].

Carrageenans are usually classified according to their structural characteristics, including sulfation patterns and the presence or absence of AnGal on the D-units. There are at least 15 different carrageenan structures [157]. The most industrially significant carrageenans are the κ, i, and λ forms. The main source of κ-carrageenan is the red algae *Kappaphycus alvarezii* [158]. Its structure is represented by alternating 3-linked β-D-galactose-4-sulfate and 4-linked AnGal units [159]. Ι-carrageenans have an additional sulfate group on the C2 (O) of the AnGal residue, which gives two sulfates per disaccharide repeating unit.

Carrageenans, as cryoprotecting agents, play a crucial role in stabilizing the structure and texture of frozen foods. In this vein, [160] evaluated the k-carrageenan functionality, used as a secondary stabilizer, in an ice cream mix. This study indicated the k-carrageenan proved to be a crucial factor for cryoprotection. The combination of k-carrageenan and xanthan gum was found to retain the texture and water-holding capacity of mashed potato for one year of frozen storage. The sensory acceptability of the treated frozen product, after 1 year, was maintained [161]. In the same way, Alvarez et al. [162] suggested that the low concentration of k-carrageenan and xanthan gum addition to frozen thawed mashed potato kept the overall acceptability. In addition, the combination gum Arabic/k-carrageenan was found to be the best cryo-protectant for the storage of beef at −18 °C [163]. Similarly, a study by Kovacevic et al. [164] confirmed the cryoprotective effect of k-carrageenan on chicken surimi during rapid freezing.

Carrageenan has long been used as a dietary supplement and is generally considered safe for humans, but recent chronic toxicological tests have shown that it may have a cumulative effect in mammals [165]. Therefore, further research is required to resolve the controversy over the safety of carrageenan.

#### 3.4.2. Algae-Containing Food: Example of Meat Products and Their Quality

Promoted by the growing interest in seaweeds due to their significant potential as functional ingredient sources, this section encloses aspects linked to the state of the art of the application of both whole algae and algae-derived compounds in meat and meat products. In 2018, Agregán et al. [166] examined the impact of seaweed (*A. nodosum*, *F. vesiculosus*, and *B. bifurcata*) extracts on the fortification of the oxidative stability of low-fat pork liver patties. During 180 days of storage at 4 °C, these authors compared studied extracts at 0.5 g∙kg^−1^ to those developed using a synthetic antioxidant (butylated hydroxytoluene (BHT) at 0.05 g∙kg^−^^1^) and untreated samples. Compared to control trials, the treated samples showed higher lipid and protein stability, kept the levels of instrumental color measured in terms of the redness (a*) and yellowness (b*), and did not modify microbial characteristics. Later, in 2019, these same authors studied the effect of *F. vesiculosus* extracts’ potency at 0.2, 0.5 and 1 g∙kg^−1^ on the shelf-life of pork patties throughout storage at 2 °C for 18 days [167]. At the end of storage, compared to untreated samples, 1 g∙kg^−1^ experiments showed lower protein oxidation parameters, expressed by thiobarbituric acid-reactive substances (TBARS) and carbonyl contents. These findings can be described by the high content of phenolic compounds, principally phlorotannin, which displayed powerful antioxidant capacity. At various concentrations (which ranged between 10% and 40%), Cox and Abu-Ghannam evaluated the incorporation of *H. elongata* seaweed extracts on chemical stability, microbial evolution, and sensory traits of cooked beef patties over 30 days [168]. The authors confirmed that enriched patties showed an increase in lipid stability, joined by good acceptance in terms of appearance, aroma texture, and taste. In an attempt to reduce nitrites in meat sausage, Sellimi et al. [169] used different levels (0.01–0.04%) of lyophilized aqueous extract from *C. barbata* seaweed. After 5 days of refrigerated storage, samples at 80 ppm of sodium nitrites reduced about 36% of the TBARS values compared to the control trials (sodium nitrites at 150 ppm). The authors assigned this protection against lipid oxidation to the existence of phenolic compounds, sterols, and fatty acids in the aqueous extract. Likewise, the red color was maintained, and turkey meat sausages were maintained during storage. The addition of edible algae Sea Spaghetti (*H. elongata*), Wakame (*U. pinnatifida*), and Nori (*P. umbilicalis*) to meat led to increases in K, Ca, Mg, and Mn levels. In addition, Nori algae increased the levels of some amino acids such as Ser, Gly, Ala, Val, Tyr, Phe, and Arg [170]. Likewise, meat fortification with algae, which supplied soluble polyphenolic compounds, increased the antioxidative potential of the whole system.

On the other hand, some attempts were made to use algae and algae-derived compounds as replacers of fat in meat products. For instance, the addition of *L. japonica* powder in the manufacture of reduced-fat pork patties was examined by Choi et al. [171]. These authors stated that the patties enriched with various levels at 1%, 3%, 5%, and 10% fat content showed a decrease in cooking loss and reductions in diameter and thickness. Furthermore, *L. japonica* powder at 1% and 3% enhanced textural parameters: springiness, hardness, gumminess, and chewiness. López-López et al. [172] reported that the addition of *H. elongata* at 5% to low-fat frankfurters enriched with n-3 polyunsaturated fatty acids (PUFA) improved the water- and fat-holding capacities, enhanced the hardness and chewiness, and lowered lightness (L*) and redness (a*) values. Nevertheless, compared to untreated samples, treated ones presented lower sensory acceptability.

With the aim to make functional and practical preservative designs, films have to obey several physico-chemical interactions. The essential operation over their production manner is the polymeric organic precipitation that might be ensured by simple and complex coacervation and gelation [173,174]. The meat industry has designed biopolymers-based materials containing algal hydrocolloids as active packaging to diminish losses and improve the shelf-life of these products.

The impact on the chemico-physio-mechanico properties and the biological potential of algal hydrocolloids films for packaging meat and derived products have recently been reported. For instance, due to its attractive properties, sodium alginate was used as a biopolymer in meat packaging. Sodium alginate showed good mechanical strength, moisture barrier, and cohesiveness. Other features such as high water viscosity, permeability, absorption capacity, and the ability to incorporate various compounds into the matrix have extended its applicability in the development of new materials in meat packaging [175,176].

Using the casting method, Puscaselu et al. [176] prepared sodium alginate/agar films utilizing glycerol as a plasticizer. After testing and identifying the best characteristics, a new film was used to package the slices of dried salami. The results indicate the possibility of substituting conventional materials with sodium alginate/agar biopolymer. The same beneficial effect of natural extracts was shown by [177] when they packed chicken breast in polymer foils with the addition of essential oils of lemon and verbena or by Kang et al. [178] when they packed low-fat frankfurters in sodium alginate film. Takma and Korel [179] produced active ethylene terephthalate (PET) films composed of chitosan and alginate as coatings incorporated with black cumin oil. The authors examined their positive impact on refrigerated chicken breast meat shelf-life. Alginate incorporation into the network matrices of the film showed anti-*S. aureus* and anti-*E. coli* activity and variations in color, pH, total viable count, and psychrotrophic bacteria counts of packaged samples.

#### 3.4.3. Drawbacks of Marine Hydrocolloids Originated from Seaweeds in Food Application

In spite of a wide range of several applications, carrageenan has some drawbacks and adverse effects on biological systems. The toxicological properties of carrageenan are the following: LD50 (rat, oral) > 5 g∙kg^−1^; LD50 (rabbit, skin) > 2 g∙kg^−1^; 4 h LC50 (rat, inhalation) > 0.93 mg∙L^−1^ [180]. Due to its sulfate group, carrageenan was revealed to have harmful effects regarding blood coagulations and immune system [124,181]. In this regard, the presence of sulfate groups on G-6 generated the strongest cytotoxicity [182,183]. In addition, the anticoagulant activity of carrageenans correlates with the contents of sulfate groups [184]. Moreover, carrageenans may induce adverse effects on human intestinal epithelial cells. For instance, the treatment of carrageenans at a range between 1 and 10 mg∙L^−1^ over 8 days was found in human colonic epithelial cells [185]. Carrageenan was also recognized to provoke an inflammatory response in the study of anti-inflammatory drugs in laboratory animals [186,187]. Recent findings demonstrated that the long-term use of carrageenans in animals resulted in ulcerative colitis or digestive system mucous layer injury and promoted tumor growth [187].

On the other hand, it should be noted that the application of alginate has significant limitations due to its macromolecular structure, poor solubility, and low bioavailability [188]. Equally, alginate can be digested chemically or enzymatically, producing alginate oligosaccharides, which have lower molecular weights and lower viscosity. Hence, it is necessary to carry out additional vital and epidemiological investigations to evaluate the hydrocolloids’ safety.

Algae are used to produce a variety of products from native and processed algomass [189]. In some regions of the world, there is a historical tradition of using certain types of algae for food purposes. Today, all countries around the world allow the consumption of the following types of algae: *A. platensis*, *A. maxima*, *C. vulgaris*, *C. pyrenoidosa*, *C. sorokineana*, and *D. salina*; regionally permitted: *N. pruniforme* (in Southeast Asian countries) and *A. flosaquae* (in the United States). Algae contain a unique complex of components required by the human body. Their cells are rich in vitamins, proteins, carbohydrates, and micro- and macroelements, not only quantitatively, but also qualitatively. For example, microscopic algae can biosynthesize 13 vitamins, whereas fish oil contains only 6 of them. The concentration of vitamins such as thiamine, riboflavin, folic acid, and provitamin A in the biomass of *C. vulgaris*, *S. elongates*, and *S. platensis* is higher than that in higher terrestrial plants. Algae of the genera *Nostoc* and *Microcystis* accumulate vitamin B12 in large amounts [190].

#### 3.4.4. Algae as a Source of Protein and Amino Acids

According to WHO data, more than 60% of the world’s population does not eat adequately, meaning they do not get enough protein from food. So, according to the conclusion of the specialists of the Russian Academy of Medical Sciences, conducting selective clinical studies throughout the country, the protein deficiency in Russia is approximately 850 thousand tons/year. Microscopic algae can solve this problem, since they contain large amounts of complete proteins essential for humans [191]. There is evidence in the scientific literature that the protein of *Chlorella*, *Scenedesmus*, *Chlamydomonos*, *Spirulina*, *Nostock* and other microalgae contains all essential amino acids—threonine, valine, phenylalanine, leucine, isoleucine, lysine, methionine, etc. In light of this, it is clear that an intensive culture of microalgae is required as an additional source of complete protein. It was demonstrated that chlorella biomass with protein contents ranging from 8 to 58 percent, carbohydrates ranging from 6 to 37 percent, and fats ranging from 4 to 85 percent can be produced by changing the cultivation conditions [192].

By varying the growing conditions, it is possible to significantly increase the yield of these and other substances (amino acids and macro- and microelements) in other types of algae.

The food industry has experience in using *S. platensis* microalgae as high-protein and vitaminized food additives, bio-dyes, as well as a biostimulants and growth regulators.

The algae cells contain significant amounts of mineral components. For example, the biomass of spirulina contains up to 528 mg/kg of iron, phosphorus—8000, potassium—14,300, magnesium—1660, manganese—22, zinc—33, and selenium—0.4 mg/kg, and it contains even more calcium than milk (up to 10,000 mg/kg). The marine unicellular red algae porphyridium is a source of carrageenin, which is used not only as an emulsifier in the food, pharmaceutical, and fermented milk industries, but also as an adhesive in leather and textile production [193].

#### 3.4.5. Use of Algae Pigments

The controlled biosynthesis of algal pigments such as chlorophylls, carotenes, xanthophylls, and phycobiliproteins is one of the most pressing tasks in biotechnology [194]. It is important that the pigments obtained from plant components are not toxic. Thus, the green alga *D. salina* is recognized as the most promising source of carotene for the biotechnological industry. It is known that under certain conditions, it can hyper synthesize carotene, the content of which in its cells can reach 10%.

A study of the *D. salina* biology and environmental factors causing its transition to the active accumulation of β-carotene in vivo showed that the biosynthesis of this compound is an adaptive response of organisms in response to extreme growth conditions, which include changes in salinity and mineral composition of the environment, temperature, and light, as well as a combination of a set of these parameters. Under industrial conditions, using the principle of separation of cell division and photosynthesis, with controlled biosynthesis of β-carotene in *Dunaliella* cells, it is possible to obtain large amounts of vitamin A precursor in short time intervals. However, the technological process of *D. salina* cultivation is still very far from ideal due to the physiological and biochemical complexity of algal metabolism and our poor understanding from the point of view of the regulation of carotene synthesis under the conditions of an industrial process.

Blue-green algae can also be a source of pigments, of which spirulina is the only microalga currently cultivated for the industrial production of these compounds. Its chlorophylls are used for coloring soaps, oils, fats, alcoholic and non-alcoholic beverages, cologne, and perfumes, and as a deodorant. In Japan, chlorophylls are used to stain fish pastes and other culinary products, and in Europe—oils, fats, aromatic essences. Food colors can be obtained from other types of algae, for example, the additional pigment phycocyanin, isolated from the blue-green alga *Phormidium* [195].

#### 3.4.6. Application of Algae in Medicine

The production of chlorophyll–carotene paste, which is the main active ingredient in the highly effective ointment “Algofin”, from microscopic algae biomass is promising for practical use. This ointment, having an antimicrobial effect on Gram-positive and Gram-negative, aerobic and anaerobic, spore-forming and asporogenic microflora, has an anti-inflammatory effect; as a result of which, it enhances the regeneration and reparation processes, thereby reducing toxicosis in patients with extensive burns, trophic disorders, and ulcers caused by radiation.

There are numerous positive results from biomedical, pharmacological, and other studies demonstrating the high efficiency of the use of algae in the treatment and prevention of a number of diseases associated with disorders of the immune, endocrine, digestive, cardiovascular, and nervous systems of animals and humans [196]. *S. platensis* has a noticeable therapeutic effect, which is determined by its unique composition: the biomass of spirulina contains easily digestible protein, free essential amino acids, a wide range of trace elements and mineral salts, polyunsaturated fatty acids, pigments, etc.

*Spirulina* preparations in the form of ointments, alcohol and oil extracts, suppositories, and tablets help to reduce blood cholesterol and the risk of obesity, reduce nephrotoxicity when exposed to heavy metals and drugs, significantly increase the population of lactobacilli and bifidobacteria in the intestine, normalizing the activity of the gastrointestinal tract, and reduce the content of blood sugar in diabetes [197]. It was found that phycocyanin, isolated from spirulina, stimulates cell growth, as well as increases immunity and resistance to cancer. This compound is one of the best radioprotectors, since it absorbs up to 40% of radioactive cesium and strontium from the human body, and its superoxide dismutase inactivates free radicals, slowing down the aging process of cells. Chlorophyll derivatives of spirulina are used for photodynamic therapy of cancer. In connection with the problem of iodine deficiency among the population of Ukraine, biotechnological methods have been developed for the production of spirulina biomass with a high concentration of iodine—up to 100 μg per 1 g of biomass, and most of the iodine is part of organic compounds that are more stable than mineral ones. All of the above facts give reasons to consider spirulina as one of the most important objects of biotechnology. The growing demand for biomass and its components led to the development of highly productive technologies for the production of *S. platensis* under controlled conditions, making it possible to formalize the entire production process of its biomass with a given biochemical composition with a high degree of accuracy.

Glycoproteins of microalgae, capable of inhibiting the growth of tumor cells, as well as carotenoids, which are antioxidants due to the presence of conjugated double bonds, bind singlet oxygen and inhibit the formation of free radicals and also have healing properties [198]. Phycobiliproteins, as additional pigments of microalgae, have found application in immunofluorescence diagnostics, where they are used as fluorescent tags. There are data indicating the possibility of using phycobiliproteins as anti-inflammatory agents. The beneficial effects of microalgae on human health, such as prebiotic, immunomodulatory, antioxidant, anticancer, and hypocholesterolemic effects, have been described in various studies; however, the mechanism providing a positive effect strongly depends on the specific strain of microalgae and the content of biologically active substances [199].

The importance of algae in medicine is also growing as regenerators of therapeutic mud and sources of obtaining unique medical preparations (blood substitutes, soluble surgical threads, and antidiabetic compounds).

One of the high-priority directions in the development of biotechnology is the search and study of new, unconventional sources of biologically active substances; the USA and Japan are leaders in this research, and the developments of scientists from France, India, Switzerland, Australia, and Argentina are also making significant contributions.

Recent data suggest that algae can be used for the targeted biosynthesis of a number of compounds. For example, *S. platensis* is capable of synthesizing iodine-containing compounds of a hormonal nature, thyroxine and triiodothyronine, which are easily digestible by the human body. The prospect of using unicellular green algae for the biosynthesis of secondary compounds such as alkaloids, steroids, and vitamins is very appealing [200].

Fucoidan is a type of polysaccharide that contains significant amounts of L-fucose groups and sulfate esters, mainly derived from brown seaweed. During the last decade, fucoidan has been widely studied due to its high biological activity. The search for new drugs has generated interest in fucoidans from microalgae [201]. During the last decade, fucoidans isolated from different types of microalgae have been widely studied regarding their anticoagulant and antithrombotic, antiviral, antitumor and immunomodulatory, anti-inflammatory, blood-lipid-lowering, antioxidant and anti-complementary properties, activity against hepatopathy, uropathy, protective effects for gastric pathology, and therapeutic potential in surgery. Compared to other sulfated polysaccharides, fucoidans are widely available from various kinds of cheap sources, which is why more and more fucoidans are being investigated in recent years for the development of drugs or functional foods [202].

Bilan et al. reported that fucoidans of brown algae *F. evanescens* C. Ag, *F. distichus*, and *F. serratus* L. consist of fucose, sulfate, and acetate [203]. *F. evanescens* C. Ag fucoidan has a linear base of alternating 3- and 4-linked 2-sulfate residues of α-1-fucopyranose: [→3)-α-1-Fuc p (2SO_3_^−^)–(1→4)-α-l-Fuc p (2SO_3_^−^)–(1→] with an additional sulfate occupying position 4 in a part of 3-linked fucose residues, while some of the remaining hydroxyl groups are acetylated randomly [189]. *F. distichus* fucoidan consists of repeating disaccharide units: [→3)-α-l-Fuc p–(2,4-di-SO_3_^−^)–(1→4)-α-l-Fuc p–(2SO_3_^−^)–(1→]. The regular structure can only be slightly disguised due to accidental acetylation and the insufficient sulfonation of several repeating units of the disaccharide [204]. Fucoidan from F. serratus has a branched structure; its main chain is →3)-α-l-Fucp–(1→4)–α-1-Fuc p–(1→, and about half of the 3-linked residues are substituted in C-4 by α-1-Fuc p–(1→4)–α-l-Fuc p–(1→3)-α-l-Fuc p–(1→trifucosidic links. Sulfate groups occupy mainly C-2, and sometimes C-4, although 3,4-diglycosylated and some terminal fucose residues can be unsulfated. Acetate groups are occupied by C-4 3-linked fucan and C-3 4-linked fucan in a ratio of about 7:3. Fucoidan also contains small amounts of xylose and galactose. The sulfated fucan from S. marginatum has a backbone of (1→4)–and (1→3)-linked-α-l-fucopyranosyl residues, which are substituted at C-2 and C-3, and fucosyl residues are sulfated mainly at C-2 and/or C-4 [204].

#### 3.4.7. Application of Microalgae in the Chemical Industry

Microalgae are also successfully used in the chemical industry. Some microalgae, for example *P. cruentum*, are used to produce resinoids, fragrant fixatives used as fragrances and dyes for food, perfumery, and other cosmetic products, as well as household chemicals. Some strains of chlorella and cenedesmus contain more than 20% resinoids in their dried biomass [191]. The main problem in the production of resinoids from marine microalgae is their high cost compared to synthetic analogs. To reduce the cost of technologies, it is possible to use waste-free production in the complex processing of microalgae biomass. Spirulina processing products are also used in cosmetology in the form of dyes, creams, emulsifiers, gelling agents, and detergents.

It should be remembered that mineral resources such as deposits of graphite, limestone, diatomites and tripoli, oil shale and gases, sapropels, some varieties of coal, and possibly oil were formed in the past geological epochs as a result of the photosynthetic activity of ancient algae, including unicellular ones.

#### 3.4.8. Application of Microalgae in Agriculture

Microalgae are widely used in agriculture. Algae of the *genera Chlorococcum*, *Spirogyra*, *Scenedesmus*, *Nostoc*, *Navicula*, *Nitzschia*, etc., are used as feed additives in livestock and poultry farming. Such additives have a pronounced positive effect: in animals—immunity increases, and their weight, fertility, and survival of juveniles increase; in birds—the size of eggs increases, and egg production and the intensity of color of egg yolk increase. Consequently, in the United States, farms for raising cattle and poultry are provided with algal ponds in which animal waste is disposed of by algae, as a result of which, 40% of the nitrogen from the wastewater is reintroduced into the algae biomass and consumed by the animals. In addition, the use of a chlorella and Scenedesmus suspension in silkworm breeding accelerates the development of silkworm caterpillars, as well as increases its viability and yields of cocoons.

The use of algae to solve the food problem, which is inextricably linked with the search for effective environmental protection methods, allows for a reduction in the anthropogenic load on terrestrial–aquatic ecosystems, which are now the primary source of food for humans and animals. Currently, the global sales of microalgae products are growing steadily: by 2028, they will reach about USD 5 billion [205].

In the context of the intensification of agricultural production and a sharp increase in anthropogenic impact on the environment, including on the soil cover, the role of biological factors in increasing soil fertility and their recultivation is significantly increasing. The skillful use and regulation of the development of soil biota, of which algae are a constant and essential component, can be of great assistance in this.

Microalgae are successfully used to increase soil fertility, that is, to replenish stocks of organic matter (such as humic acids), which helps to increase crop yields. Green (*C. vulgaris*, *S. obliquus*, *S. acutus*, *S. quadricauda*, and *S. spinosa*) and blue-green (*Nostocaceae* family) microalgae are used for this purpose. The subject of the algologization of soils was first raised when studying the stability of rice yields in India under monoculture without fertilization. It turned out that the rice fields of India are inhabited by a large number of blue-green algae, among which there are many nitrogen-fixing forms. Obviously, the use of microalgae as a biofertilizer is economically profitable and safer for the environment in comparison with chemical fertilizers [58].

#### 3.4.9. Solving Environmental Challenges with Microalgae

Another aspect of the use of microalgae in human economic life has attracted much attention recently—the ecological one. The activity of microscopic algae as utilizers of carbon dioxide can be viewed as a challenge to the 21st century. In this regard, the scale of their application will steadily expand as an alternative to solving not only technical, food, and medical problems, but also complex energy and global environmental problems [206].

Microalgae play a particularly important role in biological water purification. Considering economic efficiency, the use of algae for wastewater treatment from food industry enterprises, fish farms, livestock farms, poultry farms, and slaughterhouses is the most promising. They, as phototrophic organisms, enrich the aquatic environment with oxygen, thereby contributing to the acceleration of oxidative processes and the mineralization of organic impurities in wastewater. Algae for wastewater treatment is successfully used in the USA, Japan, and Germany. There is evidence that some blue-green algae can hydrolyze the acylanilide herbicide propanil, converting it into 3,4-dichloroaniline, which is then more rapidly destroyed by bacteria. Some cyanoprokaryotes decompose phenylcarbamate herbicides—propham and chloropropham—into aniline and chlorine derivatives. The positive role of blue-green microalgae is determined by the total effect of several significant factors: the improvement of oxygen regime due to photosynthetic aeration, the improvement of conditions for the existence of aquatic microflora, the accumulation of pollutants, and the release of biologically active metabolites. The cultivation of microalgae in wastewater, on the one hand, allows biological water purification, and on the other hand, it allows cheap biomass rich in proteins, vitamins, etc., to be obtained [207]. There is evidence that algo-bacterial cenoses contribute to the destruction of fuel oil, organic synthesis products, and other xenobiotics that enter natural water bodies as a result of human activity. The use of active strains of microorganism destructors, as well as the isolation and use of microalgae resistant to polluted waters, enabled the development of a new complex biotechnology for the purification and restoration of ecosystems contaminated with oil and oil products in water bodies and soils. These technologies enable the bioremediation of water bodies and soils that have been subjected to systematic accidental pollution with oil products and other toxicants for many years.

The cost of operating biological ponds (if appropriate land and water resources are available) per unit of treatment efficiency is 1% of the cost of the entire biological treatment. The capital cost of processing in biological ponds is 10–50% of the cost of a typical purification station.

#### 3.4.10. Application of Microalgae in Bioenergy and Space Exploration

Algae is one of the richest sources for biofuel production. The oil yield from algae is about 50%, which is significantly higher than that of rapeseed. The amount of vegetable oils produced from algae is 11,400–95,000 L∙ha^–1^, that is, massively more than from food crops [208].

In terms of potential energy yield, microalgae exceed palm oil by 8–25 times and rapeseed oil by 40–120 times, which allows them to be classified as typical representatives of vegetable oil crops.

Laboratory cultures of algae can be used to solve many fundamental problems in natural sciences. Microalgae are used as a convenient model object for elucidating the mechanisms of respiration and photosynthesis, the potential productivity of the photosynthetic apparatus, issues of biological self-regulation of the biosynthesis of various compounds, and to clarify the issues of the natural focus of some sapronous infections.

In relation to space exploration, microscopic algae, primarily chlorella, are viewed as a link in closed ecological systems capable of providing biological air regeneration and food reproduction [209]. This idea was expressed by K. Tsiolkovsky, who suggested that it is possible to use microalgae as a metabolic counterweight to humans during a long space flight or extraterrestrial settlements. Experiments have shown that in an ecosystem with closed gas and water loops, chlorella can provide a person with oxygen, absorbing carbon dioxide, and utilize the products of their vital functions for an almost unlimited time (the experiments lasted up to a year), but at the same time, a person cannot completely absorb the entire synthesized biomass of chlorella [210]. The advantages of using microalgae include the absence in the experiment of noticeable changes in the physiological and population state and side effects in the coexistence of humans and algae in closed systems, as well as high productivity (for chlorella, 25–30 L of oxygen from 1 L of suspension per day) and high the degree of reliability and stability of the algal link in providing adequate nutrition for the crew.

### 3.5. Problems Arising in the Industrial Production of Algae Products

Algae used in the food industry usually have a fishy odor of varying intensity. The volatile compounds that contribute to the smell of algae obtained by different processing methods also differ. Hydrocarbons, aldehydes, alcohols, and esters were identified in macroalgae *S. thunbergii*, *G. lemaneiformis* (*Rhodophyta*), and *S. fusiforme*. Hydrocarbons accounted for more than 60% of the total amount of components in the species *P. yezoensis* and *U. pinnatifida* (*Rhodophyta*) [211]. The fishy odor of these volatile compounds seriously affects consumer perception and limits the development of the edible algae industry; therefore, deodorization technologies need to be developed. There are methods involving the application of an acid-base salt or steam treatment. However, these methods cause some damage to algal nutrients [212]. Thus, it is necessary to develop new methods of deodorization that do not have a destructive effect on the nutrient components of algae.

Algal proteins are actively used in the food industry. Animal proteins are still the most consumed and nutritionally balanced. However, their growing demand will not be sustainable due to the low conversion efficiency and high environmental impacts of their production. Algae are considered a valuable source of proteins, but their industrial production raises several difficulties [213]:

− A high level of variability of algal proteins (protein content may vary depending on the season, temperature, and place of collection);
− The scalability of protein extraction from algae (many of the developed extraction methods are used on a small scale);
− Although algae can be a natural storage of vitamins and minerals, they can also store toxic elements such as heavy metals.

Algae polysaccharides are also of commercial value. Various industrial applications include their use as thickeners, stabilizers, emulsifiers, feedstuffs, beverages, foodstuffs, pharmaceuticals, and others [97]. Macroalgae are used for the production of hydrocolloids (for example, alginate, agar-agar, and carrageenan) [214,215]. The macroalgae of the genus *G. verrucosa* are one of the sources of agar production [98]. Marine algae are rich in carrageenans, among which, the three most commercially used are κ-, i-, and λ-carrageenans [216]. Due to their biocompatibility, exceptional physicochemical properties, as well as their ability to emulsify, thicken, gel, and stabilize, they have found several industrial applications, notably in the food industry [217].

## 4. Some Methods of Extraction of Micro- and Macroalgae Components

There is no single extraction protocol for isolating specific components from algal material, as studies involve different extraction parameters. The same extraction methods can be applied to macro- and microalgae depending on the extracted compounds, but with different methods of preliminary preparation. For example, extraction with solvents such as methanol, ethanol, water, and aqueous solutions of alcohols is most often used to obtain phenolic compounds [218]. Agregan et al. [167] described the extraction of polyphenols from brown algae *A. nodosum*, *L. japonica*, *L. trabeculata*, and *L. nigrecens*, which was carried out using microwaves.

**Extraction via maceration.** Extraction via maceration is used quite often. This method is simple and allows the temperature and pH of the extraction to be varied.

Different groups of substances can be extracted depending on the solvent used. Water-soluble proteins and R-phycoerythrin were extracted from macroalgae *M. stellatus* [219] with solutions such as tap water, pure water, 0.1 mol∙L^−1^ phosphate buffer (pH 6.5), 20 mmol phosphate buffer (pH 7.1), and 50 mmol phosphate buffer (pH 6.45). The best results were observed when extracting with 20 mmol buffer solution. Tannins, potentially possessing hemostatic properties, were isolated from *S. aquifolium* and *P. pavonica* using ethanol [220]. *P. pavonica* extract had a higher total tannin level than *S. aquifolium* extract. Sequential extraction with methanol and petroleum ether from red algae *E. cottonii* allowed steroid substances such as cholesterol, β-sitosterol, campesterol, and stigmasterol to be isolated [221]. The obtained substances demonstrated antioxidant activity, as well as toxicity towards the *A. salina* larvae.

**Soxhlet extraction.** The Soxhlet method allows the amount of solvent used to be reduced compared to maceration while achieving the quantitative extraction of components from raw materials [222]. As in the above method, different solvents are used depending on the substances to be isolated. Bio-oils, which can be used for biodiesel production, were isolated from the brown algae *S. marginatum* on a Soxhlet extractor using n-hexane [222]. Venkatesan et al. demonstrated that the Soxhlet extraction method is significantly more efficient than maceration extraction [222]. This method is applicable to microalgae as well. For example, the Soxhlet method was used to extract fatty acids from the microalgae *N. salina* [52]. N-hexane and chloroform were used as solvents. The main fatty acid compounds in the obtained extracts were palmitoleic, oleic, palmitic, cis-5,8,11,14,17-eicosapentaenoic, arachidonic, and γ-linolenic acids.

**Ultrasound extraction.** Extraction using ultrasound makes it possible to obtain a higher yield of phenolic substances in a short time in comparison with extraction via the maceration method. This is essential for the industrial production of natural antioxidants [223,224]. The study [225] used ultrasound extraction to obtain ethanol extracts from microalgae *S. obliquus*, *C. vulgaris* (*Chlorophyta*), and *S. platensis* (*Cyanobacteria*). All the extracts showed a high content of phenolic compounds, with the highest amount of phenols found in the extract of *S. platensis*, followed by *C. vulgaris* and *S. obliquus*. The results of this study were consistent with those of Ali and Doumandji [226], who reported that microalgae could produce relatively complex polyphenols. *C. vulgaris* also showed significant levels of flavonoids [227]. Alkaloids were found in all three algae. *S. platensis* and *C. vulgaris* also contained saponins. The temperature and time of extraction have a significant effect on the antioxidant activity of ultrasonic macroalgae extracts. Water–ethanol (50%) extract of *U. lactuca*, obtained via ultrasound, contained essential phenolic compounds such as quercetin [193]. The resulting extract had antioxidant activity. In this case, the optimal extraction conditions were 1 h duration and a temperature of 25 °C. Extracts obtained from *H. banksii* with 70% ethanol in an ultrasonic bath contained significant amounts of phenolic compounds with antioxidant activities towards ABTS (2,2’-azino-bis(3-ethylbenzothiazoline-6-sulfonic acid) and DPPH (2,2-diphenyl-1-picrylhydrazyl) and the ability to reduce iron ions [224]. The maximum amounts of phenolic compounds and the values of antioxidant activity were observed for extracts obtained at 30 °C for 60 min at an ultrasound power of 60% (150 W). Extraction methods using a Soxhlet apparatus, ultrasound or maceration involve the use of solvents. In studies [228,229], extraction was performed with hexane, ethyl acetate, and water. The analysis of the antioxidant activity of microalgae extracts revealed that the hexane extract of *Microchaete tenera* and the aqueous extracts of *Chlorella vulgaris*, *Fischerella musicola*, and *Fischerella ambigua* had the highest antioxidant activity. In addition, DPPH-HPLC analysis showed the high antioxidant potential of the aqueous fractions. However, from a food processing standpoint, ethanol and water are more suitable because they have GRAS status (generally recognized as safe by the US Food and Drug Administration) [229,230].

**Supercritical fluid extraction.** The supercritical extraction process has some advantages: high-speed and low-temperature extraction and contactless oxygen extraction, which allows thermolabile compounds to be obtained, and environmental friendliness [52,227]. Aliev and Abdulagatov [52] described the extraction of lipids from *N. salina* with pure CO_2_ and CO_2_ with the addition of acetone. The experimental results showed that the extraction method had little effect on the total extract yield and the fatty acid content.

The same method was used to extract biologically active compounds (fatty acids, pigments, phenolic compounds, and flavonoids) from the marine *U. clathrata*, *C. glomerata* (*Clorophyta*), *P. fucoides* (*Rhodophyta*), and their multispecies mixture), and freshwater *C. glomerata*. The content of polyphenols with antioxidant activity was approximately 2–4% [231].

Supercritical carbon dioxide is an attractive alternative to organic solvents in food extraction because it is gaseous at room temperature and pressure, which simplifies compound recovery and provides solvent-free extracts. In addition, this molecule is environmentally friendly and generally recognized as safe (GRAS) by the US Food and Drug Administration (FDA) and the European Food Safety Administration (EFSA) [232].

It was demonstrated that the yield of fatty acids was higher (Table 3) with supercritical fluid extraction than with the Soxhlet one. At the same time, the macroalgae extraction yields were much more significant than with the microalgae extraction yields.

## 5. Conclusions and Future Potential of Using Algae

The concept of functional nutrition is taking over the world and inspiring both science and industry to find innovative ingredients with physiological effects. The biological activity of food products can be increased by using algae, since their chemical components increase the nutritional value of food [191].

However, there are still some serious problems with quantifying the above benefits as well as possible side effects. Firstly, there is limited understanding of the nutritional composition of different types of algae, the influence of geographic regions and seasons, and methods of harvesting, storing, and processing, which can significantly affect the nutritional value of food products. The second problem is a quantitative assessment of the bioavailability or the proportion of functional components that actually work, depending on the time they stay in the digestive system, and assessing what factors influence the release of food components, from food preparation to genetic differentiation in the gut microbiome. The third is understanding how the nutritional and functional components of algae interact in human metabolism [45].

At present, to study the colloidal properties of algae polysaccharides, the following properties were investigated: foam-forming, surface-active, gel-forming, thickening, rheological, sorption, and compositional properties. To study the physical properties of algal polysaccharides, the following were investigated: electrical and magnetic constants, electrical resistivity, coercive force, residual induction, resistance, conductivity, capacitance, inductance per unit volume of algae, etc.

The main disadvantages of the methods for studying, processing, and using algae are the multistage and irrational use of raw materials, when some components, such as the lipid fraction, polyphenols, or fiber, become production waste. Another disadvantage of these methods is the use of toxic and expensive organic solvents for defatting biomass and obtaining preparations of lipophilic substances. The processing of algae and the production of biologically active substances using organic solvents (hexane, chloroform, etc.) can lead to environmental pollution and have a toxic effect on humans.

There are no global studies regarding the determining role of algal polysaccharides, which have the properties of enterosorbents, in the prevention of occupational and industrial health disorders. It is assumed that alginate-containing products from brown algae in the form of biogels have a similar effect, in particular, specialized food biogels from seaweed for preventive dietary and therapeutic nutrition. The analysis shows that there is a need to expand the list of substances for diets of therapeutic and prophylactic nutrition with the inclusion of alginate or alginate-containing products in it when working in hazardous working conditions.

Studies on the interactions of algal secondary metabolites in cellular systems can provide helpful information on molecular mechanisms of action and parameters such as dose requirements, efficacy, and bioavailability. Further studies on the structure–activity interactions would broaden research prospects and provide insight into the synthesis of derivatives of natural products from algae, which can be promising components for the production of pharmaceuticals [233,234,235,236].

## Figures and Tables

**Table 1:** Nutritional value of marine algae in terms of dry matter (DM).

Algae Genus and Species	Crude Proteins (%)	Total Lipids (%)	Total Carbohydrates ^1^ (%)	Ash, %	Moisture, %	Sources
*U. prolifera*	26–33 ^2^	0.20–0.80 ^2^	43–51 ^2^	9.20–25.80	5–6	[11]
*C. racemosa* var. *peltata* (*Chlorophyta*)	11	1.03	72	10.97	5	[12]
*S. oligocystum*	7–9 ^3^	3.51–5.66 ^3^	52–58 ^3^	20.34–32.45	7	[13]
*S. fusiforme* (SF-1)	9–12 ^3^	3.52–4.61 ^3^	nd	76.39–80.48	7	[14]

^1^ Calculated by dry weight; ^2^ depending on the place of collection; ^3^ depending on the season; nd—not determined.

**Table 2:** Antimicrobial activity of various algae.

Algae Genus and Species	Test Cultures against Which Algae Are Active	Sources
*G. doryphora* (R)	1–6	[117]
*U. lactuca* (C)	3, 4, 6–11	[120,121]
*E. intestinales* (C)	3, 4	[121]
*C. vagabund* (C)	3, 4	[121]
*D. spiralis* (P)	6–11	[118]
*J. rubens* (R)	6–11	[118]
*A. oryzae* (Tci)	1, 3–6, 8, 12, 13	[86]
*O. limosa* (Tci)	5, 6, 8, 13	[86]
*S. ocellatum* (Tci)	1, 5, 6, 12, 13	[86]
*T. suecica* (C)	3–6	[87]
*D. salina* (C)	4–6	[87]
*N. gaditana* (E)	3–6	[87]
*D. viridis* (C)	4–6	[87]
*P. tricornutum* (D)	4–6	[87]
*I. galbana* (H)	4–6	[87]
*C. muelleri* (D)	6	[87]
*C. vulgaris* (C)	6	[87]

1—*B. subtilis*; 2—*E. faecalis*; 3—*S. aureus*; 4—*E. coli*; 5—*P. eruginosa*; 6—*C. albicans*; 7—*S. pyogenes*; 8—*M. luteus*; 9—*S. flexneri*; 10—*V. cholerae*; 11—*A. niger*; 12—*K. pneumoniae*; 13—*S. marcescens*. P—Phaeophyceae, R—Rhodophyta, Tci—Cyanobacteria, E—Eustigmatiophyceae, H—Haptophyta, D—Diatomeae, C—Chlorophyta.

**Table 3:** Total fatty acid content (wt% in dry extract) with supercritical fluid and Soxhlet extractions.

Algae Genus and Species	Soxhlet Extraction		Supercritical Fluid Extraction CO_2_		Sources
	Solvent	Yield (%)	Solvent	Yield (%)	
*C. glomerata*	hexane	28.8 ± 0.63	CO_2_	36.4 ± 1.32	[231]
	acetone	34.0 ± 1.02			
	ethanol	21.2 ± 0.71			
*N*. salina	hexane	3.70	CO_2_	4.04	[52]
	chloroform	3.92	CO_2_^+^ acetone	4.12	

## Data Availability

The data are included in the manuscript.
